# Recombinant Porcine Interferon-α Decreases Pseudorabies Virus Infection

**DOI:** 10.3390/vaccines11101587

**Published:** 2023-10-12

**Authors:** Bowen Song, Wenkang Wei, Xueyi Liu, Yaoyao Huang, Shuaiqi Zhu, Lin Yi, Eerdunfu, Hongxing Ding, Mingqiu Zhao, Jinding Chen

**Affiliations:** 1 College of Veterinary Medicine, South China Agricultural University, Guangzhou 510642, China; songbwohno@gmail.com (B.S.); liu_xyi@163.com (X.L.); pisov2020@gmail.com (Y.H.); z17838453020@163.com (S.Z.); yilin@scau.edu.cn (L.Y.); dinghx@scau.edu.cn (H.D.); zmingqiu@scau.edu.cn (M.Z.); 2 Agro-Biological Gene Research Center, State Key Laboratory of Livestock and Poultry Breeding, Guangdong Academy of Agricultural Sciences, Guangzhou 510640, China; weiwenkang@gdaas.cn; 3 Graduate School of Agricultural and Life Sciences, The University of Tokyo, Tokyo 113-8657, Japan; eerdunfu2017@mol.f.u-tokyo.ac.jp

**Keywords:** interferon-α, pseudorabies virus, mammalian expression system, CHO-K1 cell

## Abstract

Interferon (IFN) is a cell-secreted cytokine possessing biological activities including antiviral functioning, immune regulation, and others. Interferon-alpha (IFN-α) mainly derives from plasmacytoid dendritic cells, which activate natural killer cells and regulate immune responses. IFN-α responds to the primary antiviral mechanism in the innate immune system, which can effectively cure acute infectious diseases. Pseudorabies (PR) is an acute infectious disease caused by pseudorabies virus (PRV). The clinical symptoms of PRV are as follows: reproductive dysfunction among pregnant sows and high mortality rates among piglets. These pose a severe threat to the swine industry. Related studies show that IFN-α has broad applications in preventing and treating viral diseases. Therefore, a PRV mouse model using artificial infection was established in this study to explore the pathogenic effect of IFN-α on PRV. We designed a sequence with IFN-α4 (M28623, Genbank) and cloned it on the lentiviral vector. CHO-K1 cells were infected and identified using WB and RT-PCR; a CHO-K1 cell line with a stable expression of the recombinant protein PoIFN-α was successfully constructed. H&E staining and virus titer detection were used to investigate the recombinant protein PoIFN-α’s effect on PR in BALB/c mice. The results show that the PoIFN-α has a preventive and therapeutic impact on PR. In conclusion, the recombinant protein can alleviate symptoms and reduce the replication of PRV in vivo.

## 1. Introduction

In 1957, the British scientist Lindenmann (1957) studied chicken influenza and found that inactivated avian influenza inoculated chicken embryo cells, and that the cells secreted some substances to inhibit and interfere with the virus’ replication. Scientists named this substance interferon (IFN). In 1958, the Japanese scientist Nagano also found that the same substances had a resistance to the virus and reported that the substance had a “virus inhibitory factor” [1]. Friedman discovered the antiviral mechanism of IFN in 1966, bringing people’s attention to the inhibitory effect of IFN on the virus. Subsequently, knowledge of the antiviral, anti-proliferation, immune regulation, and anti-tumor effects of IFN became widespread [2].

Due to the diverse types of IFN, each creature has different nucleic acid sequences. Natural IFN was first classified according to the source of animals: for instance, IFN from humans (HUIFN), IFN from pigs (POIFN), and IFN from dogs (CAIFN), etc. Afterward, IFN was further classified according to its antigen characteristics and physicochemical properties. In the 1960s, IFN was divided into type I and type II in accordance with its source and ability to tolerance acid [3,4]. As technology has developed in leaps and bounds, based on IFN nucleotide ranking, the role of specific receptors, and the protein’s primary structure, IFN has been categorized into types I, II, and III [5].

IFN plays an important antiviral role, affecting almost all stages of virus replication. IFN is unable to eliminate living viruses directly; however, IFN receptors that act on cell membranes induce the expression of various antiviral proteins through a series of biochemical processes in order to inhibit viruses [6,7]. When an organism’s system is stimulated by viruses, the type I interferon receptor (IFNAR) will induce the phosphorylation of STAT1 (signal transducer and activator of transcription 1) and STAT2 (signal transducer and activator of transcription 2), forming a heterodimer. This heterodimer, along with interferon-regulatory factor 9 (IRF9), forms a complex substance: interferon-stimulated gene factor 3 (ISGF3) [8]. Activated JAKs phosphorylate the tyrosine residues of the signal transducers and activators of transcription (STAT). The activated STAT dissociates from the IFNAR and enters the nucleus to regulate the expression of interferon-stimulated genes (ISGs) [9]. ISGF3 binds to the interferon-stimulated response elements (ISREs) that are upstream of ISGs, triggering their transcription. This process plays a crucial role in biological antiviral activity [10]. Additionally, STAT2 can form a complex with IRF9 and directly enter the nucleus to regulate the transcription of ISGs [11]. Type I IFN showed a better antiviral activity among the three types of IFN, especially IFN-α. IFN-α is produced by leukocytes, and its activity is stable under acidic environment conditions [12]. The homology of IFN-α in different species exceeds 70% [13]. Furthermore, IFN-α exerts pro- and anti-inflammatory effects within the cytokine network [13].

Pseudorabies (PR) is a disease caused by the pseudorabies virus (PRV), which belongs to the herpesvirus family [14,15,16]. Despite the fact that PR has been eradicated in several countries, variant strains of PRV remain prevalent in China [17]. PR is a notable disease, causing substantial economic losses for the swine industry, with pigs being vulnerable to PR. Pigs are the natural hosts of the virus, and other mammals, such as cattle, sheep, goats, cats, dogs, and raccoons, as well as wild animals, are susceptible [18,19,20].

PR causes a high mortality rate among piglets. Variable symptoms are caused by PRV, including respiratory distress, neurological symptoms, behavioral disorders, and reproductive issues. Respiratory infection is usually asymptomatic in finisher pigs, but it can cause miscarriages, a high death rate, and respiratory symptoms, like coughing and sneezing, as well as common symptoms including fever, constipation, depression, seizures, ataxia, circling, and excess salivation in piglets and mature pigs [21]. Research has determined that symptoms of PR tend to begin with intense itching, followed by neurological signs, encephalomyelitis, fever, and respiratory symptoms in many rodents and wild animals such as cattle, sheep, and rabbits [22,23]. Moreover, asymptomatic adult pigs are often susceptible to other viruses due to their compromised immune systems. This disease has caused serious harm to the swine industry. The mortality rate is close to 100% in piglets under one month old. Infections among pregnant sows may result in miscarriages or give birth to mummified, stillborn, or weakened piglets. The symptoms of boars and finisher pigs are not obvious after infection and are often ignored or misdiagnosed. The virus causes immunosuppression among adult pigs, making sick pigs susceptible to other diseases. The long-term infection of sick pigs is one of the causes of long-term infection in pig farms.

Although no specific treatment for acute infection with PRV is available, vaccination can alleviate clinical symptoms among pigs. PRV live attenuated vaccines, inactivated vaccines, and genetically engineered vaccines have all been successfully developed and put into use, effectively preventing and controlling PRV. However, these vaccines have certain disadvantages. For example, inactivated vaccines with a low immune efficiency, large doses, and occasional allergic reactions are harmful to animal health [24]. The live attenuated vaccine has specific toxicity, and there is a risk of strong virulence enhancement [25]. Subunit vaccines are expensive to produce, poorly immunogenic, and do not provide complete protection. Genetic engineering vaccines include subunit vaccines, nucleic acid vaccines, recombinant gene vector vaccines, and gene-deleted vaccines. Gene-deleted attenuated vaccines prevent and control PR in developed countries and regions such as Europe and the United States [26,27]. Other genetic engineering vaccines are under investigation.

To find an effective method for preventing or treating PRV, this study considered IFN as a critical point. In the current study, a plasmid with porcine-α4 was co-transfected with helper plasmids, PLP1, PLP2, and PLP3, into human embryonic kidney 293T (HEK-293T) cells and packaged into recombinant lentivirus. Chinese hamster ovary cells (CHO-K1) were infected with the recombinant lentivirus and monoclonal cell lines with stable expression of the recombinant protein PoIFN-α were obtained. According to in vitro and in vivo experiments, PoIFN-α effectively reduces the replication of PRV and protects infected animals from death.

## 2. Materials and Methods

### 2.1. Animals

BALB/c female mice were obtained from Sun Yat-Sen University and housed in a specified pathogen-free environment. All animal experiments complied with the Chinese government’s animal experiment regulations and the ARRIVE guidelines and were performed following the National Institutes of Health Guide for the Care and Use of Laboratory Animals.

### 2.2. DNA Construct of Porcine Interferon-α

According to the published data on porcine interferon-α4 (PoIFN-α4) in GenBank (GenBank accession number: M28623), the whole gene sequence with His-tag was optimization synthesized by Sangon Biotech named pUC-PoIF-α. The synthesized plasmid was identified using PCR and the restriction enzyme was digested with BamHI and EcoRI. Primers were designed using Oligo 7: PoIFN-α forward—5′-CCGGAATTCCGGGCCACCATG-3′; PoIFN-α reverse—5′-CGCGGATCCGCGTCAATGGTGA-3′.

The E.Z.N.A.^®^ Gel Extraction kit (Omega Bio-tek, Atlanta, GA, USA) was used to purify target DNA fragments from agarose gel PCR products. 

### 2.3. Preparation of Recombinant Lentivirus HIV-pCDH-PoIFN-α

Lentivirus vector pCDH-CMV-MCS-EF1-PURO, DH5 α, and T4 ligase were preserved at the Laboratory of Veterinary Microbiology and Immunology, South China Agricultural University. The vector pCDH-CMV-MCS-EF1-PURO and the DNA fragment mentioned in Section 2.2 were simultaneously digested with BamHI and EcoRI and the digested product was purified. Recombinant plasmid pCDH-PoIFN-α was constructed by catalyzing the joining of digested product from above. This recombinant plasmid and the helper plasmids PLP1, PLP2, and PLP3 were co-transfected into HEK-293T cells to package the recombinant lentivirus.

### 2.4. Generation of Stable Expression CHO-K1

Firstly, a concentration of puromycin that would kill almost un-transfected cells in one week was selected. The CHO-K1 cell line was infected by recombinant lentivirus and co-cultured with the selected concentration of puromycin to obtain cells that could stably express PoIFN-α protein. The cells were diluted and cloned to obtain a monoclonal cell population.

#### 2.4.1. Identification of Monoclonal Cells Using Western Blot, Immunofluorescence (IF), and Quantitative Reverse-Transcription Polymerase Chain Reaction (qRT-PCR)

The recombinant protein (HIV-pCDH-PoIFN-α) was loaded onto 12.5% sodium dodecyl sulfate-polyacrylamide (SDS-PAGE) gel. After electrophoresis, the protein was transferred to a methanol-activated polyvinylidene fluoride (PVDF) membrane. The PVDF membranes were incubated with Histidine tag mouse antibody (6x-His) (Beyotime Biotechnology, Shanghai, China) overnight at 4 °C. HRP-labeled Goat Anti-Mouse IgG (Beyotime Biotechnology, Shanghai, China), diluted with phosphate-buffered solution (PBST), was used as a secondary antibody. This was incubated with the membrane for 1 h at 37 °C. The membrane was visualized using ECL Substrate (Millipore, Burlington, MA, USA).

Positive CHO-K1 cells, incubated with ethanol, were stored at 4 °C before adding at −20 °C for 30 (min). The ethanol was decanted, and the samples were washed three times with PBS for 5 min. The permeabilized cells with the 6x-His tag mouse antibody were rinsed with PBS and stored at 4 °C in the dark overnight. The solution was rinsed, and the samples were washed with PBS three times for 5 min each. The cells were incubated with the diluted FITC-labeled Goat Anti-Mouse IgG (Beyotime Biotechnology, Shanghai, China) for 1 h at room temperature in the dark. This was then rinsed three times in PBS for 5 min each in the dark. Images were captured and samples observed through a fluorescence microscope.

An RNA sample of positive CHO-K1 cells was obtained using Trizol (Nanjing Vazyme Biotech, Nanjing, China). RNA sample, random primer, and RNase-free H_2_O were used to prepare the reaction mix for 10 min at 70 °C; then, these were bathed on ice for 2 min. Reverse transcription reagents (Takara Bio-Tech, Beijing, China) were mixed with the reaction mix to perform cDNA synthesis. Sequence-specific primers (forward—5′-CTCCATCAGAGCCGTGAGAAAG-3′; reverse—5′-GGTGCTCCTTCTTCCTCAGTCT-3′) were designed using software for qPCR. SYBR enzymes (Nanjing Vazyme Biotech, Nanjing, China) and specific primers were used in the two-step RT-qPCR process.

#### 2.4.2. ELISA Quantification of Recombinant Protein (HIV-pCDH-PoIFN-α)

Quantitative determination of porcine IFNα was performed using the pig α kg ELISA kit (MB-10063A) from Jiangsu Meibiao Biotechnology Co., Ltd. (Yancheng, China). In brief, 10 μL of cultured medium was mixed with 40 μL sample diluent and then incubated with 50 μL mixture/standard in a sample/standard well. A measure of 100 μL of HRP conjugate reagent was added to each well, covered with an adhesive strip, and incubated for 1 h at 37 °C. Repeat pumping and washing was performed for each well five times. Chromogen solution A, 50 μL, and chromogen solution B, 50 μL were added to each well. After incubation for 15 min at 37 °C in the dark, the 50 μL stop solution was added and the optical density (O.D.) was read at a wavelength of 450 nm within 15 min.

### 2.5. Identification of Toxicity of Recombinant Protein 

BHK cells were seeded into a 96-well microplate in a 5% CO_2_ incubator at 37 °C. A 4-fold serial dilution of recombinant protein was prepared, and 10 μL of the dilutant solution was added to the culture plate. Each dilution was added to 6 replicate wells on the plates. A measure of 10 μL of Cell Counting Kit-8 (CCK-8) (Beyotime Biotechnology, Shanghai, China) solution was added to each well containing cells in a 5% CO_2_ incubator at 37 °C for 1 h. The absorbance of each well was measured at a wavelength of around 450 nm using a microplate reader.

### 2.6. In Vivo Experiment

Firstly, a 50% tissue culture infectious dose (TCID50) of PRV was calculated by the Reed–Muench method on baby hamster kidney fibroblasts (BHK) cells. Then, different gradient dilutions of TCID 50 (10^2.5^ TCID50, 10^3^ TCID50, 10^3.5^ TCID50, 10^4^ TCID50, 10^4.5^ TCID50, and 10^5^ TCID50) were made for PRV-injected mice to measure the LD50 of PRV. The deaths of the mice were observed one week after injecting PRV; the median lethal dose (LD50) was calculated according to Reed–Muench. 

Forty-two BALB/c mice were randomly divided into seven groups to investigate the prevention and treatment effect of recombinant protein PoIFN-α on PR. Each mouse was intramuscularly injected with PRV venom with a proper concentration of LD50, and the IFN prevention group, the IFN treatment group, the infection group, and the negative control group were set up. The mice in the infection group were injected with PRV solution (F group). The mice in the control group were injected with normal saline (G group). For the IFN prevention group, the animals were divided into three groups wherein recombinant protein PoIFN-α was administered five (A group), three (B group), and one day (C group) before PRV infection. For the IFN treatment group, animals were divided into two groups—animals treated with recombinant protein PoIFN-α on the same day as PRV infection (D group) and animals treated the following day (E group). The survival status and pathological changes of the infected mice were observed. Symptoms of different tissues (brain, heart, liver, lungs, kidney, and spleen) were observed after dissection.

#### 2.6.1. Detection of Viral Load of PVR by qPCR 

The tissues were cut into pieces to prepare the samples. The samples were put into RNAase-free 2 mL Eppendorf with PBS and ground by beads with a homogenizer until tissues were totally cracked. The samples were pelleted at 4 °C for 5 min.

Viral nucleic acid was extracted by Viral DNA Purification Kit (Thermo Fisher Scientific Inc., Franklin, MA, USA). In light of the standard plasmid pMD-gB, kept in the laboratory, a PRV classical strain gB gene sequence primer qgB-F/qgB-R was designed using the software. The specific primer sequences were as follows:

qgB-Forward: 5′-AACAACCACAAGGTGACCGC-3′

qgB-Reverse: 5′-CGATGCAGTTGACGGAGGT-3′

The PRV loads in different mice tissues were detected using qRT-PCR and the primer was the same as qgB-F/qgB-R.

#### 2.6.2. Hematoxylin and Eosin Stain of Paraffin Sections

The tissues were obtained and stored with 4% Paraformaldehyde Fix Solution for 24 h. The paraffin blocks were processed, embedded, and sectioned by specialized equipment. The program for this process was as follows: 50% alcohol, 70% alcohol, 80% alcohol, and 90% alcohol (20 min each), anhydrous ethanol two times for 30 min, alcohol–xylene mixture (1:1) for 20 min, xylene two times for 20 min, xylene-paraffin mixture (1:1) for 30 min. The sections were embedded in melted wax to create paraffin blocks. These were then submerged in hematoxylin and the sections were rinsed in tap water. Thin sections were cut using a microtome and were dried at 45 °C. The sections were submerged in xylene for 20 min and then in an acid–alcohol solution to dehydrate; then, the sections were submerged in tap water. The next step was that the sections were immersed in Harris eosin stain for 10 min and the excess dye was rinsed with tap water. The sections were transferred to an ammonia solution for 15 min and immersed in 90% ethanol for 7 min. Finally, the sections were stained with 1% alcoholic eosin for 1 min and dehydrated through graded ethanol (70%, 95%, and 100%). A coverslip was placed on the sections using a mounting medium and the sections were observed under a microscope.

## 3. Results

### 3.1. Plasmid Identification

**Figure 1:**
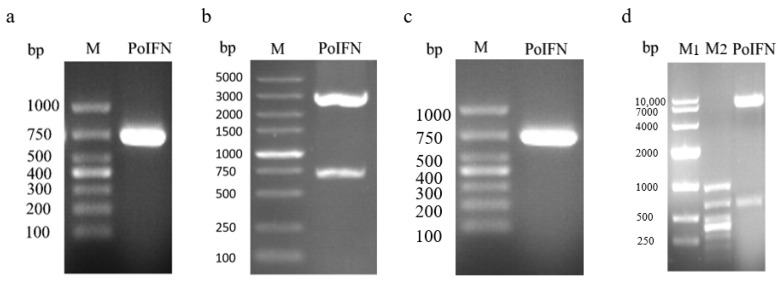
Construction and identification of pUC-PoIFN-α and HIV-pCDH-PoIFN-α. (**a**) Positive pUC-PoIFN-α plasmid identified using PCR. M: DL1000 marker. (**b**) Positive pUC-PoIFN-α plasmid identified through double digestion. The upper band is the pUC vector DNA fragment, and the lower band is the PoIFN-α DNA fragment. M: DL5000 marker. (**c**) Identification of the recombinant plasmid HIV-pCDH-PoIFN-α using PCR. M: DL1000 marker. (**d**) Identification of the recombinant plasmid HIV-pCDH-PoIFN-α through double digestion. The upper band is the lentivirus vector DNA fragment, and the lower band is the fragment of PoIFN-α DNA. M1: DL10,000 marker; M2: DL1000 bp.

pUC-PoIFN-α was synthesized using Sangon Biotech and identified using PCR. The synthesized plasmid produced a target DNA fragment of 681 bp that was present in an agarose gel (Figure 1a). The pUC-PoIFN-α recombinant plasmid was also confirmed through double digestion with BamH I/EcoR I, which produced a PoIFN-α fragment of 681 bp and a vector fragment of 3141 bp (Figure 1b). These results collectively indicated that the synthesis of plasmid pUC-PoIFN-α was successful. To obtain the recombinant plasmid HIV-pCDH-PoIFN-α, the fragment of PoIFN-α was purified from stained gel using a gel extraction kit and then connected with the lentivirus vector. The sequence of the HIV-pCDH-PoIFN-α plasmid was confirmed by PCR and double enzyme identification. A staining band at 681 bp was observed (Figure 1c) and two staining bands at 681 bp and 7384 bp, respectively, were observed (Figure 1d). These results reveal that the sequence of HIV-pCDH-PoIFN-α plasmid was correct.

### 3.2. Expression and Characterization of Recombinant Proteins

**Figure 2:**
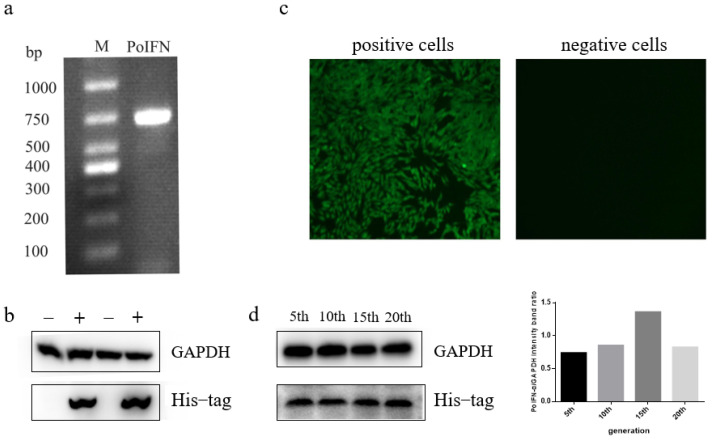
The expression of PoIFN-α gene and protein in positive CHO cells. (**a**) RNA of positive was transcribed to cDNA and then detected using PCR. M: DL 1000 bp. (**b**) Western blot analysis of recombinant protein expression in negative and positive cells. M: negative cells; CHO− K1: positive cells. (**c**) IF analysis of recombinant protein expression in positive cells and negative cells. (**d**) Western blot was performed to determine the stability of protein expression. Note: 5th, 10th, 15th, 20th—positive cells that have been continuously passaged 5 times, 10 times, 15 times, and 20 times after construction, respectively.

The obtained recombinant plasmid HIV-pCDH-PoIFN-α was co-transferred with the helper plasmid into human embryonic kidney 293T (293T) cells to attain a lentivirus solution. The CHO-K1 cell was infected by recombinant lentivirus and selected through puromycin, which exists in the vector to obtain positive CHO cells that can express PoIFN-α protein. The positive cells were confirmed through PCR and Western blot. A staining band can be observed at 681 bp in Figure 2a. The internal control protein of positive and negative CHO-K1 cells exhibited specific reactions with mouse anti-GAPDH antibodies, resulting in a single band at approximately 36 kDa. However, the Western blot results for the negative CHO-K1 cells showed that no band was observed at 25 kDa but the positive cells had one because the anti-His tag monoclonal antibodies bind to specific antigens of positive cells (Figure 2b). The original figure of identification of the recombinant protein is in Figures S1 and S2. These results indicate that positive CHO-K1 cells expressing the PoIFN-α gene were successfully obtained. After monoclonal cell screening, the selected cell lines were expanded in culture and tested using IF. It was found that the recombinant cells emitted green fluorescence, while the negative group did not show fluorescence (Figure 2c). To identify the stable expression of the protein of positive cells, the selected cells were continuously passaged and tested using Western blot. Specific bands were observed at 25 kDa in different generations, indicating stable protein-expressing cells were successfully constructed. The protein expression levels of distinct generations exhibited variation, but no significant difference exists. This might be due to the variable number of cells harvested and differences in protein degradation during the protein extraction process (Figure 2d). The original figure of stable expression of recombinant protein is in Figures S3 and S4.

### 3.3. The Concentration of Recombinant Protein PoIFNα in Positive Cells

**Figure 3:**
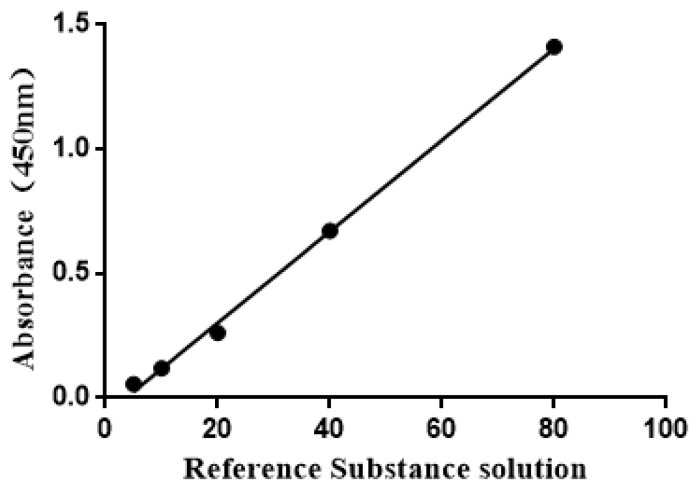
The linear regression equation of the standard curve was y = 0.0184x − 0.066, where the *y* axis represents the OD value and the *x* axis represents the concentration of the standard substance.

We created the standard curve with the concentration of the standard substance as the *X*-axis and the OD value of the standard substance as the *Y*-axis. The linear regression equation of the standard curve was y = 0.0184x − 0.066 (Figure 3), with a correlation coefficient of 0.998, which is higher than 0.99. The result reveals that the concentration of the sample calculated from the curve is highly reliable. The PoIFNα concentration was 1105 pg when the cell count was 1 × 10^6^ cells, as calculated through the regression equation.

### 3.4. Identification of Toxicity of Recombinant Protein

**Figure 4:**
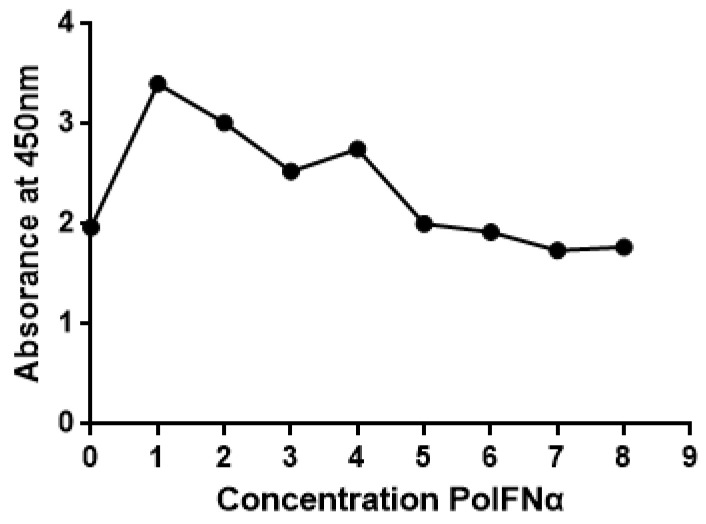
Identification of recombinant protein PoIFN-α using CCK− 8. The *y* axis represents the OD value and the *x* axis represents the recombinant protein concentration. The point of the *y* axis is the blank control group.

WST− 8 is colorless and is a yellow compound. In metabolically active cells with an intact electron transport chain in their mitochondria, cellular dehydrogenases reduce WST− 8 to a colored formazan product, resulting in a change in color in the wells. The level of absorbance presents cell viability. The toxicity of the test substance can be assessed using the OD value. A higher OD value indicates lower toxicity. The recombinant protein was diluted by a factor of 4 to confirm the toxicity of the concentration of it. The result shows that the OD values in different concentrations of recombinant protein were lower than those of the blank control group, indicating that the recombinant protein is not toxic to cells (Figure 4).

### 3.5. Calculation of TCID50 and TD50

**Figure 5:**
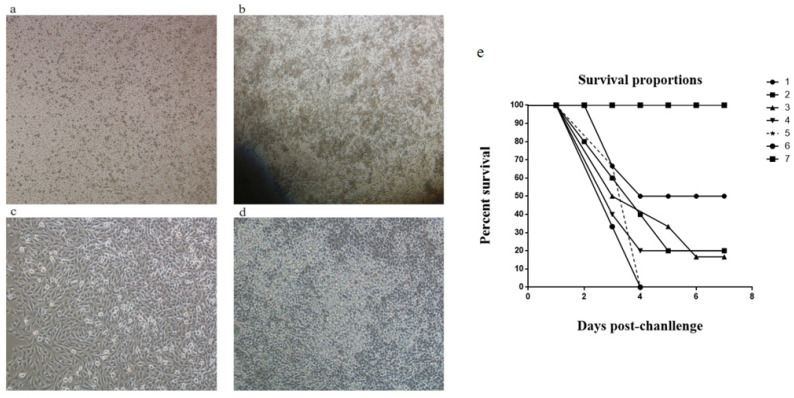
The pathological changes of BHK cells and mice survival proportion after infection. (**a**,**c**) Negative BHK cells. (**b**,**d**) The cells infected by PRV. (**e**) Mortality of mice within one week after being injected with different concentrations of PRV solution. The variety of PRV concentrations was 1: 10^4.5^ PFU/mL, 2: 10^5^ PFU/mL, 3: 10^5.5^ PFU/mL, 4: 10^6^ PFU/mL, 5: 10^6.5^ PFU/mL, 6: 10^7^ PFU/mL, 7: a control group where the mice were injected with DMEM.

The BHK cells infected with PRV exhibited a round and detached appearance, forming plaques. The cells broke apart resulting in changes in refractivity. The infected cells appeared to be more translucent than normal cells. Negative BHK cells grew in an adherent manner and the monolayer morphology of the cells remained intact, with no apparent changes (Figure 5a–d). The titer of PRV was 10^7.11^ TCID50/mL, as calculated through the Reed–Muench approach, indicating that 1 mL of the virus could infect 50% of cells after being diluted 10^7.11^ times. 

Based on the PRV titer, the virus solution’s concentration was converted to 10^8.11^ PFU/mL. A PRV virus solution was diluted into six different concentrations, and each concentration was injected intramuscularly in mice. The healthy status and mortality of the mice were observed continuously for 1 week. It was observed that the infected mice had a tendency to bite the inoculation site and had ataxia and other neurological symptoms. The survival rate of the control group in which mice were injected with DMEM was 100%. The survival rate of mice in the group infected with 10^4.5^ PFU/mL virus solution was 50% and the survival rates of other dilution groups were all below 50%, indicating that the concentration for LD50 is 10^4.5^ PFU/mL (Figure 5e).

### 3.6. The Result of the Clinical Trial

**Figure 6:**
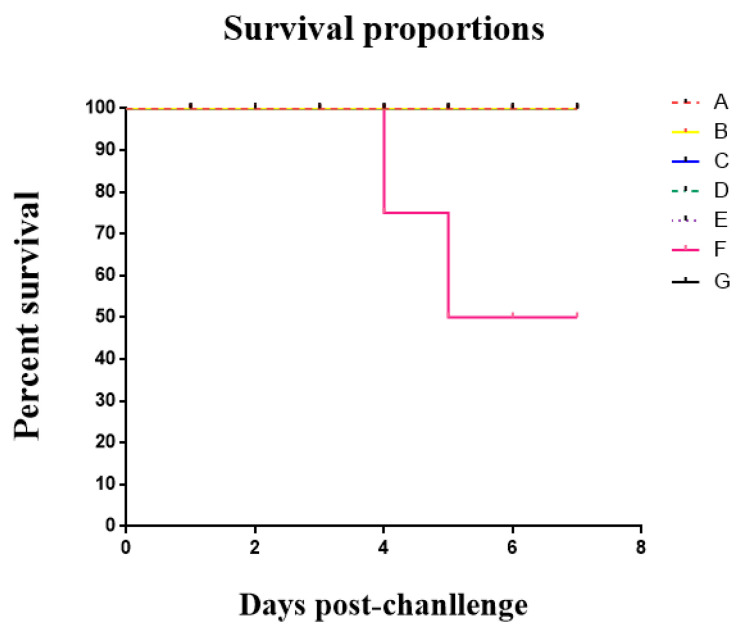
The protective effect of recombinant protein PoIFN-α on mice. A: The mice were injected with recombinant PoIFN-α 5 days before PRV injection. B: The mice were injected with recombinant PoIFN-α 3 days before PRV injection. C: The mice were injected with recombinant PoIFN-α 1 day before PRV injection. D: The mice were injected with recombinant PoIFN-α simultaneously while injection. E: The mice were injected with recombinant PoIFN-α 1 day after PRV injection. F: The mice were injected with PRV solution without any treatment. G: The mice were injected with physiological saline.

All mice injected with recombinant protein PoIFN-α survived (group A–E); however, the mice in the treatment groups (groups D and E) showed respiratory symptoms, like having heavy and fast breathing. In the infection group, the mice showed classic clinical symptoms of PR disease and three mice died by the seventh day after infection (group F). The mice in the control group did not show any symptoms. All these results indicate that the recombinant protein could alleviate the symptoms and prevent mice from death caused by PRV (Figure 6).

#### 3.6.1. Viral Load in Different Tissues of Mice

**Figure 7:**
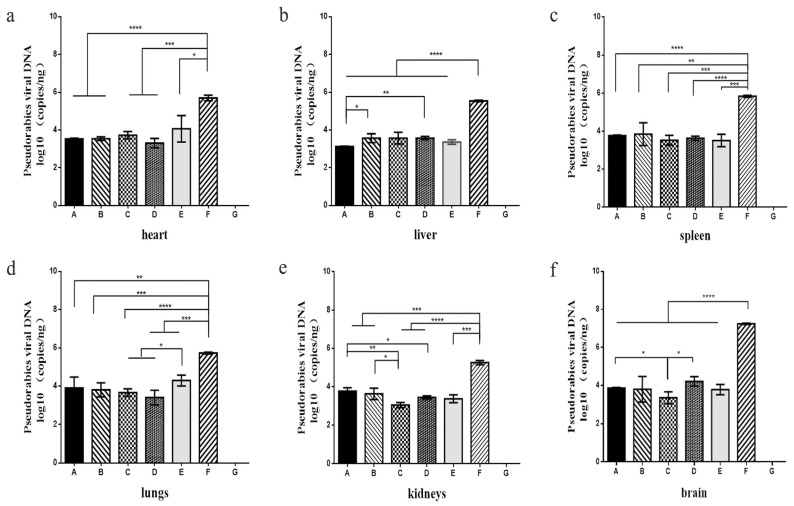
The viral copies number in different organisms. (**a**): The viral titer of the brain. (**b**): The viral titer of the liver. (**c**): The viral titer of the spleen. (**d**): The viral titer of the lung. (**e**): The viral titer of the kidney. (**f**): The viral titer of the brain. A–C groups: The prevention groups consisted of mice treated with recombinant protein PoIFN-α five (group A), three (group B), and one day (group C) before PRV infection. D and E: The treatment groups consisted of mice treated with recombinant protein PoIFN-α on the same day as PRV infection (group D) and mice treated with recombinant protein PoIFN-α on one day after infection (group E). The mice in group F were infected by PRV and in group G were injected with physiological saline. *, *p* < 0.1; **, *p* < 0.01; ***, *p* < 0.001; ****, *p* < 0.0001.

Except for the control group, PRV DNA was detected in the tissues of the mice that were infected with PRV (Figure 7). The copy numbers of viral DNA in the different tissues of the mice in the infection group (group F) were all significantly higher than those in the groups injected with recombinant protein PoIFN-α (groups A–E) (*p* < 0.05). Especially in the brain and liver tissues of the treatment and prevention groups, the viral load was significantly lower than that of the infection group. The results reveal that the recombinant protein PoIFN-α can effectively reduce the replication of PRV in different organs.

#### 3.6.2. Histopathological Sections of Mouse Tissues

**Figure 8:**
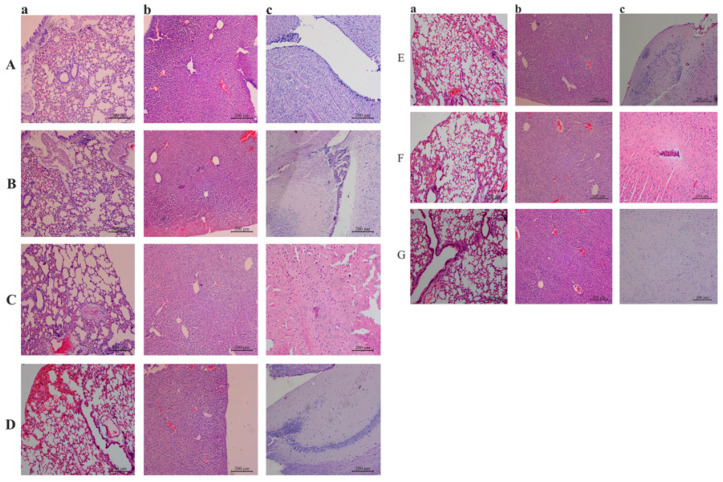
Histopathological sections of mouse tissues stained by hematoxylin and eosin. Group A–C: The prevention groups consisted of mice injected with recombinant protein PoIFN-α at different times before PRV infection, with the respective time intervals being: five days (group A), three days (group B), and one day (group C). Group D and E: The treatment groups consisted of mice treated with recombinant protein PoIFN-α on the same day as PRV infection (group D) and mice treated with recombinant protein PoIFN-α one day after infection (group E). The mice in group F were all infected with PRV and the mice in group G were injected with physiological saline. Line a: Sections of lung. Line b: Sections of liver. Line c: Sections of the brain (scare bar is 200 μm).

Histopathological sections of the lung were observed under a microscope. In the sections of the prevention groups (Figure 8 Aa, Ba, Ca), mild pulmonary hemorrhage and congestion were observed. In the sections of the treatment groups (Figure 8 Da, Ea), focal emphysema was observed with a large number of red blood cells (RBCs) in the pulmonary blebs and extracellular alveoli spaces, along with pulmonary congestion and hemorrhage, and thickening of blood vessel walls. In the sections from mice injected with PoIFN-α 1 day after infection (Figure 8 Ea) and those from the infection group (Figure 8 Fa), pulmonary atelectasis and pulmonary bullae were observed. The sections of the control group mice (Figure 8 Ga) showed normal pulmonary structure with clear alveolar structure, without significant thickening of alveolar walls and without RBCs in the pulmonary blebs and extracellular alveoli spaces.

In the liver sections of mice injected with PoIFN-α 5 days and 3 days before infection (Figure 8 Ab, Bb), there was a slight widening of the hepatic sinusoid. In the sections of the liver from mice injected with PoIFN-α 1 day before infection, on the day of infection and 1 day after infection (Figure 8 Cb, Db, Eb), there was an obvious widening of the hepatic sinusoid. A few RBCs could be observed in the perisinusoidal space, and the arrangement of hepatic cells was disordered. In the sections of the liver from the infection group mice (Figure 8 Fb), there was vacuolar degeneration in the liver. The sections of the liver from the control group mice (Figure 8 Gb) showed normal hepatic structure, with clear contours of central veins and radiating arrangement of hepatic cords along the central vein.

Histopathological sections of the brain were under a microscope. In the sections of the prevention groups (Figure 8 Ac, Bc, Cc), there were no significant pathological changes. In the sections of treatment groups (Figure 8 Dc, Ec), inflammatory cells were infiltrated in the brain. Cere edema was observed in the sections of the infection group mice (Figure 8 Fc). The sections of the control group (Figure 8 Gc) showed typical brain structures without any pathological changes.

## 4. Discussion

The biological activity of IFN can be mainly divided into four aspects. Firstly, the antiviral effect is one of IFN’s biological activity and this aspect is primarily mediated by type I IFN. During the early stages of viral invasion, type I IFN can suppress viral replication and proliferation while activating immune cells and triggering the production of antiviral proteins [28], thereby contributing to the antiviral effects. Numerous studies demonstrate that the IFN-α can effectively inhibit the replication of various viral diseases, and its species-specific nature is not evident. For example, human IFN can inhibit the replication of PRV in Vero cells [9]. Porcine IFN-α can maintain activity in various species of cells, including cattle, humans, mice, and pigs [29]. The IFN-α has a broad-spectrum antivirus capacity, thus it is used in different fields. Murine IFN-α can be used as a biological response modifier to yield significant therapeutic antitumor immune responses [30]. IFN-α subtypes could protect an animal’s cardiovascular system from viral infection [31]. Goat IFN-α exhibited prophylactic effects in response to ruminant respiratory viral infection [32]. Human IFN-α subtypes suppressed the replication of influenza A virus subtype H3N2 in human lungs [33]. Virus titers of bovine viral diarrhea virus could be controlled effectively by IFN-α [34]. Porcine IFN-α possesses broad-spectrum antiviral and high biological activity, with 1 mg of recombinant porcine IFN-α containing billions of active units. It is confirmed that porcine IFN-α exhibits antiviral activity similar to natural interferons and shows immune regulation. This study designed a sequence including porcine IFN-α sequence (M28623), Serumalbumin preproprotein signal peptide, and a 6x His tag; the sequence was inserted into a lentivirus vector to obtain a recombinant plasmid.

Expression systems can normally be categorized into prokaryotic and eukaryotic systems. Each expression system has its own specific characteristics. For instance, prokaryotic expression systems are a low-cost option and yield a high expression level. Moreover, the productions are easy to separate and purify. However, the produced proteins are often present in the form of inclusion bodies, requiring multiple steps such as denaturation, refolding, and purification to restore protein activity [35]. Eukaryotic expression systems can be subdivided into yeast, insect baculovirus, and mammalian cell expression systems. Yeast cells are rapidly reproduced, making them suitable for large-scale industrial production. They are also considered safe for laboratory use and the purification process is relatively straightforward. However, the protein products are easy to degrade, and the expression levels can be challenging to control [36]. Conversely, baculovirus can accommodate high levels of protein and provide proper folding and post-translational modifications. It is also safe and easy to manipulate. However, the insect system typically results in transient expression, requiring a large amount of virus replication which has higher costs [37]. Mammalian cells are commonly used in the eukaryotic expression system. Although they require a long period of production and have higher costs, proteins expressed in mammalian cells have long-term effectiveness. Mammalian cells provide correct folding of complex proteins and perform a wide range of post-translational modifications [38,39]. CHO cells are widely employed as host cells in the industrial production of recombinant protein therapeutics [40]. CHO cells can produce high-quality biological products that meet clinical requirements and express ectopic proteins similar to animal proteins. Based on experimental conditions, a mammalian expression system was chosen to express the recombinant IFN protein.

Lentiviral infection permanently integrates therapeutic transgenes into the chromosomes of target cell [12]. Lentiviral vectors are typically based on HIV-1 and share similar features, such as their spherical shape [41]. Lentiviral vectors have some advantages, such as a high transfection efficiency, a wide range of infectivity, and the capacity to carry larger exogenous genes [42,43]. They are less likely to induce host immune reactions. Lentiviral vectors are increasingly utilized in animal research and have shown promising results. Lentiviral vectors have significantly enhanced transfection efficiency, stability, and safety through multiple modifications and improvements [44,45,46,47]. In this study, the goal is to obtain a mammalian cell line that can express recombinant PoIFN-α protein stability. The plasmid carrying the PoIFN-α gene was cloned into the pCDH-CMV-MCS-EF1-Puro lentiviral expression vector and co-transfected with the helper plasmids PLP1, PLP2, and PLP3 into HEK-293T cells to package the recombinant lentivirus. The obtained virus infected the CHO cells to enable the stable expression of the target protein. The results of this study show that the protein produced by CHO cells can be expressed stably. The recombinant protein is not toxic to normal cells. This means that a large amount and a long-term expression of recombinant protein can be produced for in vivo experiments. 

PRV poses a significant threat to the swine industry as an acute infectious disease. Due to the silent infection and long-term carrier status of finisher pigs, PRV has proven difficult to eliminate completely. Mutated strains of PRV pose a greater threat compared to the classical strains. Currently, there is no effective treatment for PRV; vaccines play a preventive role by reducing the economic losses caused by PRV in the swine industry [48]. In this experiment, a mouse model of PRV artificial infection was established to investigate the effect of recombinant protein PoIFN-α on PRV. The results of pathological dissection showed that the recombinant protein could effectively alleviate the symptoms caused by PRV in mice. Quantitative viral load results indicated that the viral copy numbers in the infection group were higher than those in the group with recombinant protein, revealing that the recombinant protein could effectively suppress viral replication in vivo. While PRV can infect a variety of animals, it currently has a great impact in the livestock industry. Therefore, the next step will be to study the effects of recombinant proteins on the virus in pigs.

## 5. Conclusions

This study constructed a recombinant plasmid containing an IFN gene and a cell line that can stably express IFN proteins; the produced recombinant proteins are safe and appropriate for their purpose. The in vivo experiment showed that the recombinant PoIFN-α protein effectively prevents and treats PR, improves PR symptoms in infected animals, and inhibits virus replication. This study indicates the potential of this recombinant protein for the prevention and treatment of viral diseases, providing data-based support and a theoretical basis for the future development of mammalian-cell-expressed IFN-α antiviral drugs.

## Data Availability

No new data were created or analyzed in this study.

## Supplementary Materials

The following supporting information can be downloaded at: https://www.mdpi.com/article/10.3390/vaccines11101587/s1, Figure S1: Identification of recombinant protein; Figure S2: Internal reference for the identification of recombinant protein; Figure S3: Identification of stable expression; Figure S4: Internal reference for the identification of stable expression.
