# Supplementary material for: Recombinant Porcine Interferon-α Decreases Pseudorabies Virus Infection
Source: Vaccines (Basel). 2023 Oct 12;11(10):1587. doi: 10.3390/vaccines11101587 (PMC10610829; doi:10.3390/vaccines11101587)
Supplement: Supplementary file 1 [file vaccines-11-01587-s001.zip › vaccines-2600934-supplementary.pdf]

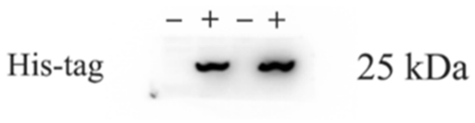

Figure S1. Identification of recombinant protein.

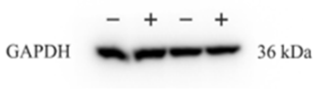

Figure S2. Internal reference for the identification of recombinant protein.

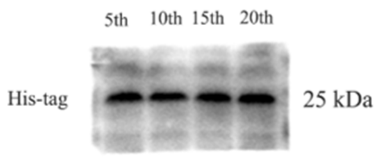

Figure S3. Identification of stable expression.

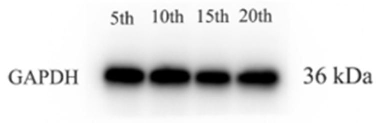

Figure S4. Internal reference for the identification of stable expression.
